# Ultrasound evaluation of schistosomiasis-related morbidity among the Xakriabá people in the state of Minas Gerais, Brazil

**DOI:** 10.1590/0100-3984.2019.0047

**Published:** 2020

**Authors:** Carolina Coimbra Marinho, Aline Joice Pereira Gonçalves Nicolato, Vivian Walter Reis, Rosiane Cristina dos Santos, Jaime Costa Silva, Henrique Pereira Faria, George Luiz Lins Machado-Coelho

**Affiliations:** 1 Universidade Federal de Minas Gerais (UFMG), Belo Horizonte, MG, Brazil.; 2 Universidade Federal de Ouro Preto (UFOP), Ouro Preto, MG, Brazil.; 3 Ministério da Saúde, Fundação Nacional de Saúde (Funasa), Brasília, DF, Brazil.

**Keywords:** Schistosomiasis mansoni, Population groups, Ultrasonography

## Abstract

**Objective:**

To use ultrasound to investigate the morbidity related to schistosomiasis in the Xakriabá indigenous population.

**Materials and Methods:**

This was a field-based census study conducted in the territory of the Xakriabá people. A total of 166 individuals were invited, and 148 (≤ 77 years of age) agreed to participate. Most participants underwent abdominal ultrasound, physical examination, and stool examination. Mann-Whitney U and chi-square tests were used for comparisons. We determined risk by calculating odds ratio (OR) and performed logistic regression analysis.

**Results:**

*Schistosoma mansoni* eggs were found in 31 (26.7%) of the 116 stool samples examined, 22 (70.9%) of the 31 being from individuals 4-16 years of age. The median count was 144 eggs/g of feces (interquartile range, 264). Of the 105 participants examined with ultrasound, 68 (64.8%) had hepatomegaly (left lobe), 6 (5.7%) had splenomegaly, and 4 (3.8%) had portal hypertension. Egg-positive stool samples were more common in those with an enlarged left lobe (OR = 3.4; 95% confidence interval (CI): 1.1-11.2; *p* = 0.043). Periportal fibrosis was found in 30 participants (28.6%), of whom 9 (30%) had pattern C, 10 (33.3%) had pattern D, and 11 (36.7%) had pattern Dc. Age was the only independent risk factor for fibrosis (*p* = 0.007). Fibrosis was up to nine-fold more common in alcohol drinkers than in nondrinkers (OR = 9.28; 95% CI: 2.60-33.06; *p* < 0.001). Among the 138 participants in whom the clinical form was classified, the chronic hepatic form was identified in 54 (39.1%), of whom 32 (59.2%) were under 30 years of age and one (1.8%) was hepatosplenic.

**Conclusion:**

Schistosomiasis in the Xakriabá population is characterized by a high frequency of egg-positive stool samples, predominantly in children/adolescents, and by chronic hepatic form in the young, especially among alcohol drinkers.

## INTRODUCTION

The territory of the Xakriabá people is the largest demarcated indigenous area in the state of Minas Gerais, Brazil, where conditions are favorable for the transmission of schistosomiasis, because the vector is present and there is poor sanitation^(1)^. A previous parasitological survey^(2)^ showed ongoing transmission of *Schistosoma mansoni* in two rural villages within the Xakriabá territory-Dizimeiro and Peruaçu-near the Peruaçu river, in 2017. The villages are situated within the municipality of São João das Missões, 677 km north of the capital city of Belo Horizonte. At that time, 15% of the individuals screened tested positive and were treated with antiparasitic drugs. However, liver and spleen involvement were not studied.

In cases of schistosomiasis, examination of the liver and spleen is crucial to recognizing the risk of complications, such as upper gastrointestinal bleeding secondary to variceal rupture^(3)^. Liver biopsy, the gold-standard diagnostic method for periportal fibrosis (PF), is not routinely used because it is invasive^(4)^. In contrast, abdominal ultrasound is noninvasive, practical and safe. It is now considered the best imaging technique to detect changes suggestive of chronic schistosomiasis in the field^(5)^. Nevertheless, it is a dynamic and examiner-dependent method^(6)^. The Niamey-Belo Horizonte protocol outlines the World Health Organization (WHO) standard recommendations for ultrasound in schistosomiasis^(7)^. According to the protocol, if some degree of periportal thickening is observed, the image is compared with standard imaging patterns of liver parenchyma. Patterns range from A, indicating normal parenchyma, to F, characterized by highly echogenic, often conglomerated, bands extending from the main portal vein and its bifurcation to the liver surface, which may be retracted. Pattern B is characterized by diffuse echogenic foci; pattern C shows echogenic rings corresponding to the pipe-stem fibrosis seen in a perpendicular scan; and patterns D and E are characterized by progressive echogenic thickening and patches extending around the portal bifurcation and the main stem. Mixed patterns can also be seen^(7)^. However, it should be borne in mind that the intensity of fibrosis on ultrasound may not correlate with disease severity^(8)^.

The objective of this study was to investigate positivity for schistosomiasis and parasite burden, as well as the frequency of liver and spleen involvement on ultrasound, in the Xakriabá population. We also attempted to determine whether the chronic forms correlate with clinical and epidemiological factors.

## MATERIALS AND METHODS

A descriptive cross-sectional field-based study was conducted to investigate the frequency and morbidity related to schistosomiasis in Xakriabá indigenous population. All 166 residents of Dizimeiro and Peruaçu were invited; 148 (89%) participated. Participants 5-and-older had epidemiological, clinical, parasitological, and ultrasound data recorded. Children under 5 had parasitological examination only.

This work is part of a study designed to assess the health of indigenous populations in Minas Gerais, approved by the National Research Ethics Council (Ruling no. 902/2006; Registration no. 12827) and by the National Indigenous Foundation (Authorization no. 73/CGEP/06). Permission was also obtained from local indigenous leaders and from the local council for the health of indigenous populations. Participants who tested positive for intestinal parasites were treated with antiparasitic drugs. All health abnormalities were officially reported to local authorities.

### Parasitology

To diagnose infection with *S. mansoni*, we employed the TF-test stool test (Bio-Brasil Biotecnologia, Anápolis, Brazil). Approximately 1 g of feces was examined in a set of three tubes containing preservative solution (10% formalin) processed with 3 mL of ethyl-acetate and a drop of neutral detergent. The tubes were connected to a centrifuge tube and spun at 1500 rpm for 1 min (Elektra GoldLine; Laborline, São Paulo, Brazil). The supernatant was discarded, and the pellet was resuspended in distilled water, after which it was examined by light microscopy at magnifications of ×10 and ×40 (3 slides per sample). This method was chosen in order to simplify the logistics in the field. Samples were collected in preservative solution and transported to the research laboratory for analysis. Participants testing positive for *S. mansoni* were invited to provide a fresh sample for quantitative analysis by the Kato-Katz method^(9)^, with a commercially available kit (Kato-Katz CoproKit; Campinas Medical, Campinas, Brazil). Stool samples (standard weight, 41.7 mg each), collected in a plastic vial, were processed, washed for 30 min, and examined by light microscopy (at ×10 and ×40, 3 slides per sample). Samples with viable *S. mansoni* eggs were labeled as positive. The parasite burden, defined as the number of eggs per gram (eggs/g) of feces, was calculated by multiplying the mean egg count of each slide by 24. Infection was graded, as recommended by the WHO^(10)^, as mild (1-99 eggs/g), moderate (100-399 eggs/g) or intense (≥ 400 eggs/g). The presence of eggs of other parasites was recorded.

### Ultrasound

**Figure 1:**
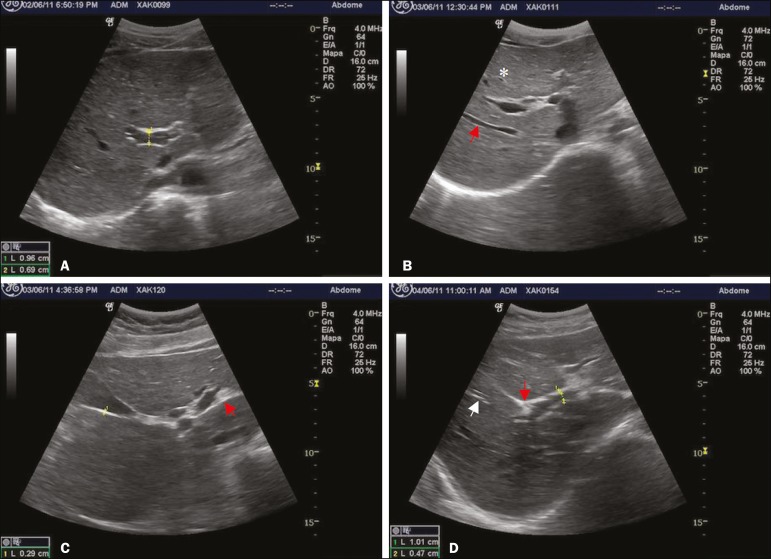
Liver parenchyma patterns. **A:** Pattern B, starry sky. **B:** Pattern C, rings (asterisk) and pipe-stem fibrosis (arrow). **C:** Pattern D, a ruff around the portal bifurcation (arrow). **D:** Pattern Dc, mixed pattern with a ruff (red arrow) and pipe-stem fibrosis (white arrow).

A trained radiologist, who was blinded to the parasitological and clinical findings, used a portable ultrasound system (Logiq i; GE Healthcare, Chalfont St. Giles, UK) with a multifrequency (2.5-5 MHz) convex transducer. Participants ≥ 5 years of age underwent fasting ultrasound in accordance with the WHO guidelines for ultrasound in schistosomiasis^(7)^. Measures were adjusted for height and classified as normal or increased. Hepatomegaly, splenomegaly, and portal hypertension (PH) were diagnosed when the left lobe of the liver, spleen, and portal vein measures, respectively, were above adjusted reference values. The reference measure for gallbladder wall thickness was < 3 mm for persons weighing ≤ 30 kg and < 4 mm for those weighing > 30 kg^(5)^. The reference measure for the internal diameter of the splenic and superior mesenteric veins was < 9 mm for both^(11)^. A diagnosis of PF was made when ultrasound images of the liver were consistent with the WHO patterns C, D, E, F, or any combination of those^(7)^. As previously described^(12)^, the PF patterns were grouped for analysis as “absent” (A or B) or “mild”(C, D, or Dc). Figure 1 shows images characteristic of the standard imaging patterns of fibrosis.

### Clinical forms

Identification of the chronic forms of schistosomiasis was based on the parasitological and ultrasound findings. Hepatointestinal schistosomiasis was defined as a positive TF-test result, with or without PF on ultrasound, and no PH or splenomegaly. The hepatosplenic form was defined as PF with splenomegaly, with or without PH on ultrasound. Participants with PF on ultrasound and a negative or unavailable stool test result were classified as having chronic schistosomiasis because of the high local rate of positivity^(13)^. Participants with a negative or unavailable stool test result and without PF were classified as not having schistosomiasis.

### Epidemiology

We collected data related to demographic characteristics, contact with natural waters, previous treatment for schistosomiasis, risk factors for chronic liver diseases, and the physical examination. Alcohol drinking was assessed as “yes” or “no”.

### Statistical analysis

The IBM SPSS Statistics software package, version 22.0 (IBM Corp., Armonk, NY, USA) was used for data storage and analysis. Normal distribution was verified by the Shapiro-Wilk test. Continuous variables are presented as median and interquartile range (IQR); the Mann-Whitney U test was used for comparisons. Categorical variables are presented as absolute and relative frequencies; the chi-square test was used for comparisons. For risk analysis, we calculated odds ratios (ORs). A logistic regression model was used for the multivariate analysis. Variables with significance level of 0.2 were included in the multivariate model. The level of significance was set at 0.05.

## RESULTS

### Clinical findings

We evaluated 148 individuals ≤ 77 years of age, with a median age of 14 years (IQR, 25 years). Of the 148 participants, 70 (47.3%) were women. Table 1 presents the descriptive statistics of the epidemiological, physical examination, and ultrasound findings. In all cases in which the liver was palpable, it had a normal consistency and a smooth edge. No ascites, abdominal collateral circulation, jaundice, deterioration in general health, or palpable spleen were detected. There were no statistical associations between positivity for Schistosoma eggs and the clinical findings.

**Table 1:** Descriptive statistics of residents of Xakriabá territory (n = 148) in the state of Minas Gerais, Brazil.

Variable	Valid data*	N	(%)
Years of schooling			
< 1	114	14	(32.5)
1-8	114	69	(60.5)
> 9	114	8	(7.1)
Occupation			
Teacher/health care worker	111	4	(3.6)
Homemaker	111	11	(9.9)
Farmer	111	26	(23.4)
Student	111	36	(32.4)
Risk factors for chronic liver disease			
Blood transfusion	124	3	(2.4)
Gastrointestinal bleeding	123	38	(30.9)
Alcohol drinking	122	51	(41.8)
Contact with natural waters	110	110	(100.0)
Treatment for schistosomiasis	80	58	(72.5)
Overweight/obese	120	24	(20.0)
Palpation of the liver			
Palpable left lobe	104	15	(14.4)
Palpable right lobe	104	16	(15.4)
Enlarged features† (median, IQR)			
Left liver lobe (8.7 cm, 2.8)	105	68	(64.8)
Spleen (8.6 cm, 1.7)	105	6	(5.7)
Portal vein diameter (7.8 mm, 2.6)	105	4	(3.8)
Gallbladder wall thickness (2.4 mm, 0.8)	105	1	(0.9)
Liver fibrosis pattern (7)			
C	30	9	(30.0)
D	30	10	(33.3)
Dc	30	11	(36.7)

* Total number of individuals for whom valid data were obtained.

† On abdominal ultrasound.

### Parasitology

Stool samples were available for 116 (78.4%) of the 148 participants. Among those 116 participants, positivity for any parasite was identified in 104 (89.0%) and *S. mansoni* was identified in 31 (26.7%). The ages of the 31 individuals testing positive for *S. mansoni* ranged from 4 to 31 years, and 22 (70.9%) were between 4 and 16 years of age. Of the 31 participants with schistosomiasis, 15 (48.4%) had an egg-positive stool sample and provided a fresh sample for quantitative analysis. Three of those 15 samples were found to contain no eggs. Egg counts ranged from 0 to 384, with a median of 144 (IQR, 264).

### Ultrasound

**Figure 2:**
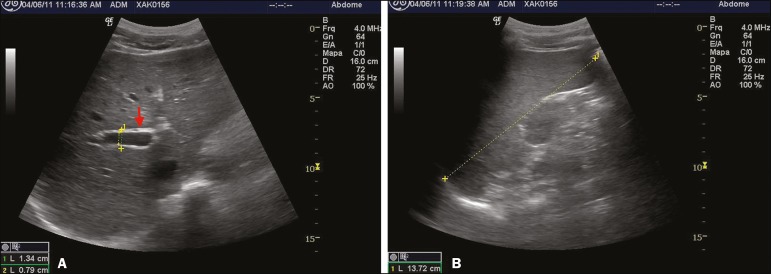
**A:** PF pattern D (arrow): periportal thickening without PH (measures: 1. portal vein outer diameter, dashed line 1, 1.34 cm; and 2. portal vein inner diameter, dashed line 2, 0.79 cm). **B:** Spleen enlargement (dashed line 1, 13.72 cm).

Ultrasound records were available for 105 (70.9%) of the 148 participants. Of those 105, 30 (28.6%) had PF. Fibrosis patterns C, D, and Dc were detected. Of the 30 participants with PF, 8 (26.5%) were in the 10- to 19-year age group and 20 (66.7%) were in the 40- to 49-year age group. The risk of PF was tenfold higher in the ≥ 30-year age group than in the < 10-year age group (OR = 10; 95% confidence interval (CI): 2.0-50.4; *p* = 0.001). Neither an egg-positive stool sample nor previous treatment for schistosomiasis was found to be associated with splenomegaly or PF, nor was PF found to be associated with PH or splenomegaly. One participant had splenomegaly and the Dc pattern of PF, without PH (Figure 2).

There were no significant differences between the participants with and without an egg-positive stool sample, in terms of the ultrasound measures. However, an egg-positive stool sample was 3.4 times more common in those with an enlarged left lobe of the liver (OR = 33.4; 95% CI: 1.1-11.2; *p* = 0.043).

Of the 148 participants, 4 (2.8%) had PH. Three of those four had an egg-positive stool sample. There was no significant difference between those with and without PH in terms of the median age-11.5 (IQR, 20) vs. 17 (IQR, 25); *p* = 0.267. No collateral circulation was detected in any of the participants.

Alcohol drinkers were at a 3.5-fold greater risk of having PF (OR = 3.54; 95% CI: 1.4-8.8; *p* = 0.009, data not shown). When stratified by age, the risk of PF was nine times higher in alcohol drinkers ≥ 30 years of age than in nondrinkers < 19 years of age (Table 2).

**Table 2:** Prevalence and risk of PF by age group and alcohol drinking, among residents of Xakriabá territory (n = 103) in the state of Minas Gerais, Brazil.

		PF							
		No			Yes				
Alcohol drinking	Age (years)	N	(%)		N	(%)	OR (95% CI)		*P*
No	5-19	30	(83.3)		6	(16.7)	1		—
	20-29	4	(80.0)		1	(20.0)	1.25	(0.12-13.24)	0.85
	≥ 30	10	(83.3)		2	(16.7)	1.00	(0.17-5.77)	1.00
Yes	5-19	16	(76.2)		5	(23.8)	1.56	(0.41-5.90)	0.51
	20-29	6	(66.7)		3	(33.3)	2.50	(0.48-12.88)	0.26
	≥ 30	7	(35.0)		13	(65.0)	9.28	(2.60-33.06)	< 0.001

For 83 (56%) of the 148 participants, ultrasound and parasitological results were both available for multivariate modeling. Age, alcohol consumption, PH, and enlarged left lobe of the liver, adjusted for gender and egg positivity, were included in the risk analysis for PF (*p* < 0.2 for all). Age remained the only independent risk factor (*p* = 0.007). No independent risk factors for PH were detected.

It was possible to classify the clinical form of schistosomiasis in 138 (93.2%) of the 148 participants. Among those 138 individuals, chronic forms were identified in 54 (39.1%), of whom 32 (59.2%) were under 30 years of age-median age, 15 (IQR, 25)-and 53 (98.1%) had the hepatointestinal form, the remaining individual, a 21-year-old man, having the hepatosplenic form.

## DISCUSSION

To our knowledge, this was the first field-based study using ultrasound to describe schistosomiasis-related morbidity in an indigenous population living in demarcated lands in Brazil. The Xakriabá land is situated in an endemic area targeted by the Brazilian National Schistosomiasis Control Program^(14)^. The fact that 89% of the residents participated in the present study indicates the level of concern on the part of the population and local leaders. We detected a high (26.7%) frequency of egg-positive schistosomiasis and a high (39.1%) prevalence of the chronic forms. Despite the low frequency of the hepatosplenic form (1.8%), the high frequency of the hepatointestinal form (38.4%), together with the young age of affected subjects, implies a high risk of developing the severe form of the disease^(15,16)^. Positivity for any parasite was an alarming 89%, and polyparasitism was common (data not shown). Also remarkable is the high frequency of alcohol drinking at ages as young as 10 years.

The 26.7% rate of positivity for Schistosoma eggs in this survey, performed in 2011, was more than three times higher than the annual national averages reported between 1990 to 2010^(17)^. This finding is consistent with those of a previous study involving the same population^(2)^. Four years after the mass treatment of children and adolescents, the transmission cycle continues, indicating a failure to improve local sanitation. A few previous studies have reported the frequency of schistosomiasis in other indigenous peoples in Brazil, all in Minas Gerais. The rate of positivity for schistosomiasis among the Maxakali people was reported to be 23.7% in 2009^(18)^ and 51.9% in 2013^(19)^; among the Pataxó people, it was reported to be 5.0% in 2012^(20)^. No liver or spleen involvement was reported in any of those studies.

In the present study, an egg-positive stool sample was more common in children 4-16 years of age. This finding is in agreement with those of a previous study involving the nonindigenous population of another endemic area in Minas Gerais^(16)^. The distribution of positivity by age in endemic areas typically shows higher frequencies among preschool- and school-age children than among adolescents and adults. Therefore, school-age children have been the target of schistosomiasis control programs^(21)^. Current guidelines recommend mass treatment of school-age children in areas where positivity is above 25%^(17)^.

In our sample, parasite burdens were moderate. However, this finding must be interpreted with caution because only 15 samples (3 slides each) were quantitatively assessed. Therefore, the parasitic burden might have been underestimated, given that increasing the number of samples has been shown to increase the accuracy of the method^(22)^. One study employing the same method achieved a sensitivity of 59.7% by examining at least 2 slides of 3 samples per person^(23)^.

Periportal fibrosis on ultrasound in subjects living in endemic areas indicates a high probability of schistosomiasis^(8)^. If the appropriate guidelines are followed, ultrasound is a highly accurate method for diagnosing schistosomiasis-related morbidity^(5)^. However, differential diagnoses should be considered^(11)^. In the present study, the frequency of PF was 28.3%. Although chronic viral hepatitis was not ruled out, we identified no changes related to other chronic liver diseases, such as cirrhosis, steatosis, and cysts. Because the study population lived in an endemic area with early, recurrent exposure to infection, the identification of PF by ultrasound, even if the stool sample tested negative, was strongly suggestive of schistosomiasis^(6)^. In addition, we found no association between PH and splenomegaly. This is in keeping with the findings of a previous study showing that the intensity of PF may not correlate with the clinical profile of the patient^(8)^. Other causes of PH and splenomegaly, such as portal vein thrombosis and visceral leishmaniasis, were not investigated in our survey. Additional diagnostic workup would be useful to exclude other diagnoses.

We found that an egg-positive stool sample and PF both showed a positive correlation with age, a finding consistent with lifelong recurrent infections, as described by others^(15,16)^. Similar results were obtained in a comparative study of schistosomiasis conducted in Egypt and Kenya^(15)^. Studies conducted in Mali^(24)^ and Tanzania^(25)^ reported that PF was identified in 8% of children 5-9 years of age. That indicates that transmission is established and stable in the area, as well as that PF begins in early childhood.

The assessment of PF, PH, hepatomegaly, and splenomegaly, as recommended by the WHO, rely on quantitative and qualitative information^(7)^. Regarding PF, quantitative assessment of the thickness of portal vein wall, particularly in the secondary branches, proved to be impractical. It has been considered too time-consuming, as well as being complex, and has been shown to have low reproducibility^(5)^. Findings of enlarged secondary branches did not add clinically relevant information in our study.

Enlargement of the left lobe of the liver has long been recognized as a characteristic of hepatosplenic schistosomiasis^(26)^. This finding is usually ascribed to increased blood flow to the left lobe due to PH^(27)^. However, we found that an enlarged left lobe of the liver did not correlate with PH, although it did correlate with an egg-positive stool sample. That suggests that the migration of eggs into small portal tributaries produces a similar increase in blood flow to the left lobe in the hepatointestinal form, even before development of full hepatosplenic form.

Evaluation of the liver by imaging methods has been the subject of various recent studies in the radiology literature of Brazil^(28-32)^. The availability of affordable, good quality ultrasound machines has allowed the development of focused point-of-care ultrasound protocols applicable to tropical diseases. Ultrasound evaluation of schistosomiasis has proved practical and clinically meaningful^(5,6,16)^. Although full application of the WHO protocol might be complicated and time-consuming, a simplified point-of-care ultrasound protocol with a few standardized liver and spleen views could help detect severe disease and determine the risk of complications. The presence of PF pattern D, E, or F would indicate the need for screening of esophageal varices by endoscopy. The diagnosis of PH, hepatomegaly, and splenomegaly can be made by simple measures in standard views^(33)^.

In the present study, alcohol drinking was positively associated with PF and represented an independent risk factor for liver changes, especially in individuals over 30 years of age. Unquantified alcohol consumption was also identified as a risk factor for PF in Tanzania^(25)^. In a study involving the nonindigenous population of another endemic area in Minas Gerais^(34)^, alcohol abuse, defined as consumption > 60 g/day, was associated with periportal thickening in schistosomiasis. Doppler ultrasound might be useful in making the differential diagnosis between schistosomiasis-related and alcohol-related cirrhosis. Azeredo et al.^(35)^ reported a positive association between maximal flow velocity in the portal vein and hepatic artery peak systolic velocity in patients with schistosomiasis-induced PF, whereas that association was negative in patients with cirrhosis. However, Doppler ultrasound failed to identify the hepatic artery in over 60% of the patients with PF in that study.

We detected chronic schistosomiasis in nearly 40% of the individuals in our sample. The hepatosplenic form was identified in only one individual, corresponding to a prevalence of 1.8%, which is lower than the 10% typically reported in endemic areas^(16,34)^. However, that case occurred in an individual under 30 years of age. Participants with the hepatointestinal form were also young and are therefore at high risk of developing the hepatosplenic form in the future. A retrospective study conducted in Minas Gerais after the implementation of schistosomiasis control programs found an overall decrease in morbidity. In that study, the median age of the individuals with the hepatosplenic form was 45 years^(14)^. That underscores the urgent need for disease control to protect new generations from this incapacitating, life-threatening disease.

### Limitations

Our study has some limitations. The failure to undertake a complete differential diagnostic workup for chronic liver diseases, especially viral hepatitis, was a major limiting factor. Serological analysis would have been useful in resolving diagnostic uncertainty. However, because blood collection was not foreseen before ethics approval, it could not be performed. Another limitation was that alcohol intake was not quantified. Although it might have allowed more detailed inferences on the alcohol and PF association, alcohol intake is often subject to memory bias. Additionally, alcohol drinking is usually taboo among indigenous populations.

## CONCLUSION

Among the Xakriabá people, the rate of positivity for schistosomiasis was higher than the national average. Moderate parasite burdens were detected (median 144 eggs/g). Egg-positive stool samples were most common among school-age children, and PF was found in children and adolescents. Age was the only independent risk factor for PF, and alcohol drinking was significantly associated with the development of liver fibrosis. These findings suggest that the Xakriabá people are not given sufficient attention in official programs designed to reduce morbidity related to schistosomiasis.
